# Evaluation of ultrasonographic approaches aimed at determining distinct abdominal adipose tissue depots

**DOI:** 10.20945/2359-3997000000584

**Published:** 2023-01-17

**Authors:** Nadja Fernandes da Silva, Cláudia Porto Sabino Pinho, Alcides da Silva Diniz

**Affiliations:** 1 Universidade Federal de Pernambuco Departamento de Nutrição Recife PE Brasil Departamento de Nutrição, Universidade Federal de Pernambuco (UFPE), Recife, PE, Brasil; 2 Hospital das Clínicas, Universidade Federal de Pernambuco Pronto-Socorro Cardiológico de Pernambuco, Universidade de Pernambuco Departamento de Nutrição Recife PE Brasil Hospital das Clínicas, Universidade Federal de Pernambuco (HC-UFPE); Pronto-Socorro Cardiológico de Pernambuco, Universidade de Pernambuco (Procape UPE); Departamento de Nutrição, UFPE, Recife, PE, Brasil

**Keywords:** Abdominal obesity, abdominal circumference, visceral adipose tissue, cardiometabolic risk

## Abstract

**Objective:**

To analyze different anatomical sites in the abdominal region, in order to determine the positional parameter that identifies a higher level of visceral adipose tissue (VAT) and confers a greater cardiometabolic risk.

**Materials and methods:**

This is a methodological study in which VAT was evaluated by ultrasonography (USG) in three anatomical sites in the abdomen, while the abdominal circumference (AC) was measured using seven different protocols. Additionally, the glycemic and lipid profile, C-reactive protein, and the presence of systemic arterial hypertension were evaluated.

**Results:**

One hundred and six individuals with an average age of 42 (36.8-46.2) years were included. The evaluation of the calibration of the ultrasound procedure for the analysis of VAT by intra- and inter-evaluators showed high reproducibility. The pattern of abdominal fat distribution differed between sexes, with higher mean VAT in males (p < 0.05) and higher mean SAT (subcutaneous adipose tissue) in females (p < 0.005). In the abdominal scan applied to women, higher levels of VAT and lower levels of SAT were observed in the narrower waist region, between the iliac crest and the last rib (p < 0.001). In males, the profile of adipose disposition along the abdomen was uniform (p > 0.05). Correlations between VAT measured by USG and cardiometabolic parameters were relatively stronger in the upper abdomen (p < 0.05).

**Conclusion:**

Women accumulate more VAT in the narrower waist region, while men accumulate VAT uniformly across the abdomen. There was relative superiority in predicting cardiometabolic risk in the upper abdomen for both sexes.

## INTRODUCTION

Abdominal obesity is characterized by the accumulation of excess subcutaneous (SAT) and visceral adipose tissue (VAT), representing an important cardiovascular and metabolic risk factor ( 1 ). The interest in measuring intra-abdominal adiposity has grown increasingly due to the recognition of VAT as an adipose compartment that is metabolically active and involved in the development of health complications ( 2 ).

Imaging methods are considered reference procedures for the evaluation of abdominal adiposity for allowing to determine subcutaneous fat separately from visceral fat ( 3 ). Among them, USG has been showing great potential, as besides being less costly, more accessible, and more secure ( 4 ) compared to other methods, it also allows determining body compartments in different sections, providing a better understanding of the distribution of SAT and VAT ( 5 ). However, studies still seek to standardize the ideal anatomical site for measuring the different abdominal adipose tissue compartments using this method ( 6 ).

Among anthropometric measurements, waist circumference (WC) is recognized as the doubly indirect inference method for evaluating central adiposity that can be better associated with VAT ( 7 ), as well as with cardiometabolic complications ( 8 ). As it is presented as a *proxy* for abdominal adiposity, its validity depends on the degree of correlation with reference methods ( 9 ).

Although several studies have reinforced the safety, validity, and ease of using AC as a predictor of visceral adiposity, there is still no consensus on the anatomical site where this anthropometric parameter should be measured ( 10 ). The lack of methodological standardization to obtain this measurement can compromise the comparison between results of different studies and its use as an instrument in clinical practice, besides causing the values obtained to be under- or overestimated, which may lead to a potential error in the interpretation of its results ( 11 ).

Furthermore, the fact that this measure is used as an index of central obesity, being recommended by several health organizations as an instrument for tracking the risk of metabolic and cardiovascular diseases, reinforces the need to establish and standardize the most appropriate anatomical site for predicting cardiometabolic risk ( 12 ).

In this context, the present study aims to determine the positional parameter of the abdominal region that identifies higher levels of VAT and confers a higher cardiometabolic risk.

## MATERIALS AND METHODS

This is a methodological study carried out between 2020 and 2021 involving adults of both sexes recruited among health professionals from a public hospital in Northeastern Brazil, being approved by the ethics committee of the Ethics and Research Committee on Human Beings of the University of Pernambuco (UPE) and approved under protocol number 271.400/2013. The procedures employed in the study are in accordance with the ethical standards for human experiments.

The sample was built based on voluntary adhesion, consisting of health professionals of a public hospital in northeastern Brazil, aged between 20 and 60 years, of both sexes. Individuals with physical limitations and clinical conditions that made it impossible to carry out the anthropometric and abdominal adipose tissue evaluations, such as individuals with hepatomegaly and/or splenomegaly, ascites, recent abdominal surgery, and/or who had undergone surgical treatment for weight loss, pregnant women, and those who had children up to 6 months prior to the screening for the study were excluded.

To calculate the sample size, an *α* error of 5%, a *β* error of 20%, a mean correlation between the AC measurement and the VAT obtained by USG in young women of 0.45 (p), and a variability of 0.15 (d^2^) ( 13 ) were taken into consideration, with an estimated minimum *n* of 87 individuals. To correct eventual losses, this number was increased by 25%, totaling a sample size ( *n* )of 109 individuals.

Information on age, sex (male and female), and skin color were collected. Individuals aged between 20 and 40 years were considered young adults, while mature adults were considered as individuals aged between 40 and 59 years ( 14 ). Skin color was self-defined by the respondent, who should select between white, brown or black ( 15 ).

For the variable alcohol consumption, the classification of excessive alcohol consumption was used considering the estimate quantified by the I Brazilian Guidelines on Cardiovascular Prevention (>30 g/day for men and >15 g/day for women) ( 16 ). The individuals were classified as smokers, non-smokers or ex-smokers. Individuals who smoke at least one cigarette a day were classified as smokers; individuals who had never smoked were classified as non-smokers; and individuals who smoked at some point in their lives, but not in the last six months prior to the survey were classified as ex-smokers ( 17 ).

The physical activity level was determined by the International Physical Activity Questionnaire ( 18 ) in its short version. A physical activity score below 150 minutes per week was used to classify individuals as insufficiently active or sedentary ( 19 ).

Hypertension was determined when the participant reported a previous diagnosis issued by the physician, the use of antihypertensive drugs, and/or presented the diagnosis in their clinical record.

Among biochemical analyses, the following parameters were evaluated: fasting plasma glucose (FPG) and glycated hemoglobin (HbA1C), lipid profile (triglycerides, total cholesterol, and fractions), and the inflammatory status, evaluated by C-reactive protein (CRP).

Blood glucose and lipid profile were analyzed by the enzymatic method, while HbA1c and conventional CRP were analyzed by turbidimetry. Biochemical analyses were carried out using a Cobas Integra 400® analyzer (Roche Diagnostics) in the Laboratory of Clinical Analyses in the service.

The BMI was obtained through the following equation: Weight/Height². The individuals were classified as with or without excess weight according to the cutoff limits recommended by the World Health Organization ( 20 ).

AC was measured in duplicate and repeated when the measurement error was greater than 0.1 cm using an inelastic flexible measuring tape with accuracy of 0.1 cm, directly on the skin, in a horizontal plane around the abdomen in the seven following different regions: 1) in the narrower region between the iliac crest and the last rib (AC1) ( 21 ); 2) Immediately below the bone landmark of the last rib (AC2) ( 22 ); 3) in the midpoint between the last rib and the iliac crest (AC3) ( 23 ); 4) 1 cm above the umbilical scar (AC4) ( 24 ); 5) at the umbilical scar level (AC5) ( 25 ); 6) immediately above the bone landmark of the iliac crest (AC6) ( 26 ); and 7) in the region of largest abdominal circumference (AC7) ( 27 ).

VAT and SAT were evaluated by USG using a Vivid T8 Pro Color Doppler Ultrasound (GE, P.O., Asia) machine, with USG being performed by a single observer trained according to the study protocol. Participants were evaluated in the supine position with the right arm raised after fasting for at least four hours ( 28 ). The convex electronic transducer at a frequency of 3.5 MHz and the linear transducer at a frequency of 6.0 MHz were positioned transversely in order to perform a longitudinal scan of the xiphoid process to the umbilicus along the linea alba ( 5 ), having as reference for the measurements the following external landmarks: 1) the narrower region between the iliac crest and the last rib ( 21 ); 2) the midpoint between the last rib and the iliac crest ( 29 ); and 3) 1 cm above the umbilical scar ( 30 ).

Visceral fat thickness was considered as the greatest distance, in centimeters, between the internal (deep) surface of the rectus abdominis muscle and the anterior aortic wall, in which the reference limits of 9 cm and 8 cm were used for classifying high levels of VAT in men and women, respectively ( 31 ). Subcutaneous fat thickness was considered as the distance in centimeters between the skin and the anterior surface of the linea alba ( 4 ).

The measurements were evaluated in duplicate and repeated when the measurement error was greater than 0.1 cm, with the individual at expiration, and without exerting pressure on the abdomen, in order to not underestimate the result ( 4 ).

Data were entered into the software Epi-info, version 6.04, and statistical analysis was performed using the software Statistical Package for Social Sciences (SPSS), version 22.0.

Initially, the reproducibility of the USG measurements of intra- and inter-evaluators was evaluated in a percentage of 10% of the calculated sample size, adopting the intraclass correlation coefficient and a limit of agreement of 95%, with analysis of measurements being performed in triplicate for each anatomical site. An evaluator that is trained and experienced in the evaluation of body composition by ultrasound was considered as a reference for the calibration of the evaluator of this study.

Then, exploratory data analysis and outlier exclusion were performed. Continuous variables were tested for normality using the Kolmogorov-Smirnov test. The data of variables that presented Gaussian distribution were expressed as mean and standard deviation. Variables with non-Gaussian distribution were presented as medians and their respective interquartile ranges.

The Student’s t-test for independent samples was used to compare means between groups, while one-way ANOVA was used to compare USG and AC measurements at different anatomical sites, using the Bonferroni test *a posteriori* .

Pearson’s or Spearman’s correlation was used to evaluate the degree of relationship between VAT, which was measured by imaging exam (USG), doubly indirect inference (AC), and biochemical parameters. The correlation strength was interpreted as weak (r < ±0.4), moderate (r ranging from ±0.4 to ±0.7), and strong (r > ±0.7). The significance level of 0.05 was adopted for all statistical analyses.

## RESULTS

A previous evaluation of the reproducibility of intra- and inter-evaluator USG measurements was performed in a percentage of 10% of the sample, showing high inter-evaluator reproducibility and an Intraclass Correlation Coefficient (ICC) greater than 0.97 for VAT and greater than 0.99 for SAT; intra-evaluator reproducibility was equally high, with ICC greater than 0.9 for all VAT and SAT evaluations.

One hundred and nine individuals were included in the study, and after eliminating losses due to lack of data or inconsistency of information, 106 individuals comprised the final sample. The average age was 42 (36.8-46.2) years, with predominance of females (73.6%, CI95%: 64.5-81.0) and a higher rate of brown individuals (49.1%, CI95%: 39.7-58.4). The prevalence of systemic arterial hypertension (SAH) was 19.8% (CI95%: 13.3-28.4), overweightness was verified in 67.9% (CI95%: 58.6-76.1) of individuals, and 28.3% (CI95%: 20.6-37.5) of the individuals presented VAT values above the reference limit of the classification of cardiovascular risk ( Table 1 ).

**Table 1:** Sample characteristics of adult health professionals, stratified by sex (n = 106)

Variables		Males n% (CI_95%_)		Females n% (CI_95%_)		p-value *
Age group						0.677
	Young adults	12	24.5 (26.5-60.9)	37	75.5 (36.7-58.4)	
	Mature adults	16	28.1 (39.1-73.5)	41	71.9 (41.6-63.3)	
Skin color						0.014
	White	3	9.1 (3.71-27.2)	30	90.9 (28.4-49.6)	
	Black	9	42.9 (17.9-50.7)	12	57.1 (9.0-25.0)	
	Brown	16	30.8 (39.1-73.5)	36	69.2 (35.5-57.1)	
Arterial hypertension						0.802
	No	22	25.9 (60.5-89.8)	63	74.1 (70.7-88.0)	
	Yes	6	28.6 (10.2-39.5)	15	71.4 (12.0-29.3)	
Alcohol consumption						0.271
	No consumption	13	21.0 (29.5-64.2)	49	79.0 (51.7-72.7)	
	Consumption	12	32.4 (26.5-60.9)	25	67.6 (22.8-43.0)	
	Excessive consumption	3	42.9 (3.7-27.2)	4	57.1 (2.0-12.5)	
Smoking						0.078
	Non-smokers	22	24.2 (60.5-89.8)	69	75.8 (79.5-93.8)	
	Smokers	3	75.0 (3.7-27.2)	1	25.0 (0.2-6.9)	
	Ex-smokers	3	27.3 (3.7-27.2)	8	72.7 (5.3-19.0)	
Physical activity						0.133
	Sedentary	11	36.7 (23.6-57.6)	19	63.3 (16.2-34.9)	
	Active	17	22.4 (42.4-76.4)	59	77.6 (65.1-83.8)	
Nutritional Status						0.837
Malnutrition		0	0 (0.0-12.1)	1	100 (0.2-6.9)	
Eutrophy		10	30.3 (20.7-54.2)	23	69.7 (20.5-40.4)	
Overweight		9	23.1 (17.9-50.7)	30	76.9 (28.4-49.6)	
Obesity		9	27.3 (17.9-50.7)	24	72.7 (21.6-41.7)	
Visceral adipose tissue						0.310
	Normal	18	23.7 (45.8-79.3)	58	76.3 (63.7-82.7)	
	Excessive	10	33.3 (20.7-54.2)	20	66.7 (17.3-36.3)	

* Pearson Chi Square.

CI95%: confidence interval of 95%; without excess weight: BMI < 25 kg/m^2^; with excess weight: BMI ≥ 25 kg/m^2^; VAT normal: < 9 cm for men and < 8 cm for women; VAT excessive: ≥ 9 cm for men and ≥ 8 cm for women.

The pattern of abdominal fat distribution differed between sexes for all anatomical sites measured in the abdomen, with higher mean VAT for males (p < 0.05) and higher mean SAT for females (p < 0.005). In the abdominal scan applied to women, higher concentrations of VAT and lower concentrations of SAT in the narrower waist region between the iliac crest and the last rib (p < 0.001) were observed. In males, the profile of adipose disposition, as well as the mean ACs showed uniformity along the abdomen (p > 0.05 and p = 0.564) ( Tables 2 and 3 , respectively).

**Table 2:** Comparative analysis of the averages of Visceral Adipose Tissue (VAT) and Subcutaneous Adipose Tissue (SAT) evaluated by ultrasound in three anatomical sites in the abdominal region of adult individuals, according to sex (n = 106)

Variables	Total (n = 106)	Males (n = 28)	Females (n = 78)	p-value *
VAT1 (cm)	7.4 (±2.1) a	8.2 (±2.8)	7.1 (±1.8) a	0.042
VAT2 (cm)	6.5 (±2.2) b	7.4 (±2.8)	6.1 (±1.9) b	0.031
VAT3 (cm)	6.1 (±2.2) b	7.1 (±2.9)	5.7 (±1.8) b	0.016
p-value **	<0.001	0.329	<0.001	

Variables	Total (n = 106)	Males (n = 28)	Females (n = 78)	p-value *
SAT1 (cm)	2.5 (±1.1) a	2.0 (±1.1)	2.7 (±1.0) a	0.004
SAT2 (cm)	3.1 (±1.2) b	2.5 (±1.2)	3.3 (±1.1) b	0.002
SAT3 (cm)	3.2 (±1.3) b	2.6 (±1.3)	3.5 (±1.2) b	0.002
p-value **	<0.001	0.158	<0.001	

* Student’s t-Test for independent samples.

** one-way ANOVA.

a,b Different letters mean statistical differences by the Bonferroni test.

VAT1: the narrower region between the iliac crest and the last rib; VAT2: the midpoint between the last rib and the iliac crest; VAT3: 1 cm above the umbilical scar; SAT1: the narrower region between the iliac crest and the last rib; SAT2: the midpoint between the last rib and the iliac crest; SAT3: 1 cm above the umbilical scar.

**Table 3:** Comparative analysis of the averages of seven anatomical sites of measurement of the abdominal circumference (AC) in adult individuals, according to sex (n = 106)

Variables	Total (n = 106)	Males (n = 28)	Females (n = 78)	p-value *
AC1, cm (mean/SD)	87.1 (±12.1) a	92.8 (±14)	85.0 (±10.8) a	0.003
AC2, cm (mean/SD)	88.8 (±12.9) a,b	94.6 (±14.9)	86.8 (±11.5) a	0.006
AC3, cm (mean/SD)	92.3 (±13.4) b,c,d,e	97.1 (±16.1)	90.6 (±12.0) b	0.061
AC4, cm (mean/SD)	95.2 (±13.8) c,d,e,f	98.5 (±16.9)	94.0 (±12.4) b,c	0.201
AC5, cm (mean/SD)	96.9 (±13.9) d,e,f	99.1 (±16.6)	96.1 (±12.8) c,d	0.321
AC6, cm (mean/SD)	97.4 (±13.8) e,f	99.3 (±16.7)	96.8 (±12.6) c,d,e	0.397
AC7, cm (mean/SD)	99.5 (±13.9) f	100.2 (±16.8)	99.3 (±12.9) d,e	0.765
p-value **	<0.001	0.564	<0.001	

* Student’s t-Test for independent samples.

** One-way ANOVA.

a,b,c,d,e,f Different letters mean statistical differences by the Bonferroni test.

AC1: the narrower region between the iliac crest and the last rib; AC2: immediately below the bone landmark of the last rib; AC3: midpoint between the last rib and the iliac crest; AC4: 1 cm above the umbilical scar; AC5: at the umbilical scar level; AC6: immediately above the bone landmark of the iliac crest; AC7: in the region of largest abdominal circumference.

In women, the smallest abdominal perimeters were identified in the upper part of the abdomen (AC1 and AC2), while the largest circumferences were identified in the umbilical scar (AC5, AC6, and AC7) (p < 0.001) ( Table 3 ).

Men expressed very strong correlations between the three VAT measurements and all AC measurements at the seven anatomical sites of the abdomen, with all correlation coefficients above 0.9 (r > 0.9; p < 0.001). Among women, a strong correlation was also observed between the AC and VAT measurements obtained from all anatomical sites, although with greater variation and a slightly lower performance (correlation coefficient ranging from 0.75 to 0.85). The highest correlation coefficients were expressed between VAT measurements and the AC measurements measured in the upper abdomen, with the worse performance observed in the region with the largest abdominal circumference (AC7) ( Table 4 ).

**Table 4:** Pearson’s correlation (r) between three measurements of Visceral Adipose Tissue (VAT) and Subcutaneous Adipose Tissue (SAT) evaluated by ultrasound, with averages of abdominal circumference (AC) measured in seven anatomical sites in adult individuals, according to sex (n = 106)

Variables	Males					
	VAT1	VAT2	VAT3	SAT1	SAT2	SAT3
AC1	0.935 *	0.914 *	0.902 *	0.672 *	0.673 *	0.677 *
AC2	0.930 *	0.916 *	0.902 *	0.691 *	0.693 *	0.699 *
AC3	0.925 *	0.909 *	0.900 *	0.695 *	0.699 *	0.703 *
AC4	0.933 *	0.917 *	0.905 *	0.703 *	0.703 *	0.706 *
AC5	0.934 *	0.918 *	0.907 *	0.704 *	0.703 *	0.707 *
AC6	0.934 *	0.916 *	0.905 *	0.715 *	0.711 *	0.716 *
AC7	0.934 *	0.919 *	0.910 *	0.710 *	0.703 *	0.707 *

Variables	Females					
	VAT1	VAT2	VAT3	SAT1	SAT2	SAT3
AC1	0.834 *	0.851 *	0.815 *	0.753 *	0.744 *	0.761 *
AC2	0.833 *	0.849 *	0.818 *	0.767 *	0.757 *	0.772 *
AC3	0.848 *	0.858 *	0.827 *	0.742 *	0.751 *	0.761 *
AC4	0.807 *	0.835 *	0.783 *	0.712 *	0.756 *	0.768 *
AC5	0.821 *	0.845 *	0.800 *	0.728 *	0.759 *	0.766 *
AC6	0.828 *	0.845 *	0.816 *	0.727 *	0.749 *	0.750 *
AC7	0.773 *	0.797 *	0.749 *	0.725 *	0.762 *	0.768 *

* p < 0.001.

AC1: the narrower region between the iliac crest and the last rib; AC2: immediately below the bone landmark of the last rib; AC3: midpoint between the last rib and the iliac crest; AC4: 1 cm above the umbilical scar; AC5: at the umbilical scar level; AC6: immediately above the bone landmark of the iliac crest; AC7: in the region of largest abdominal circumference; VAT1: the narrower region between the iliac crest and the last rib; VAT2: the midpoint between the last rib and the iliac crest; VAT3: 1 cm above the umbilical scar; SAT1: the narrower region between the iliac crest and the last rib; SAT2: the midpoint between the last rib and the iliac crest; SAT3: 1 cm above the umbilical scar.

Regarding the correlation between SAT and AC measurements, it was observed that the correlations were influenced by the region measured in men, with strong correlations being evidenced when the AC was evaluated in lower regions of the abdomen (r > 0.700; p < 0.001) and moderate correlations being evidenced when the AC was evaluated in the upper regions of the abdomen (r < 0.7; p < 0.001). In females, this difference was not detected and all measurements were strongly correlated, regardless of the positional parameter adopted in the measurement (r > 0.700; p < 0.001) ( Table 4 ).

Correlations between VAT and SAT measurements and biochemical variables were more intensely expressed in men, with strong and inverse correlation being evidenced between the BMI and VAT measurements in the upper regions of the abdomen (VAT1 and VAT2) (r = -0.73 and r = -0.72; p < 0.05) and a strong positive correlation being evidenced between CRP and all three VAT measurements (r >0.7; p < 0.05). SAT was only moderately correlated with CRP and exclusively when measured in the region delimited at 1 cm above the umbilical scar (r = 0.64; p = 0.007). For women, most of the correlations found were moderate ( Table 5 ).

**Table 5:** Pearson’s (r) or Spearman’s ( *rho* ) correlation between three measurements of Visceral Adipose Tissue (VAT) and Subcutaneous Adipose Tissue (SAT) evaluated by ultrasound with biochemical parameters in adult individuals, according to sex.

Variables	Males (n = 17)					
	VAT1	VAT2	VAT3	SAT1	SAT2	SAT3
FPG b	0.123	0.123	0.096	-0.361	-0.056	-0.054
HbA1C b	0.627 *	0.669 *	0.613 *	-0.367	-0.190	0.201
TG b	0.620 *	0.627 *	0.505 *	-0.258	-0.061	0.139
TC a	0.481 *	0.500 *	0.430	-0.110	-0.041	0.017
HDL-c a	-0.729 *	-0.721 *	-0.673 *	-0.327	-0.381	0.348
LDL-c a	0.566 *	0.544 *	0.489 *	0.047	0.092	0.132
CRP b	0.718 *	0.753 *	0.782 **	0.212	0.456	0.643 *

Variables	Females (n=62)					
	VAT1	VAT2	VAT3	SAT1	SAT2	SAT3
FPG b	0.224	0.311 *	0.270 *	0.205	0.170	0.208
HbA1C b	0.333 *	0.411 *	0.381 *	0.229	0.207	0.196
TG b	0.326 *	0.332 *	0.347 *	0.256 *	0.237	0.212
TC a	0.219	0.218	0.241	0.143	0.079	0.049
HDL-c a	-0.362 *	-0.328 *	-0.336 *	-0.115	-0.166	-0.178
LDL-c a	0.267 *	0.267 *	0.289 *	0.085	0.060	0.031
CRP b	0.279 *	0.378 *	0.261	0.250	0.265	0.234

* p < 0.05.

** p < 0.001.

a Pearson’s correlation.

b Spearman’s correlation.

FPG: Fasting Plasma Glucose; HbA1C: glycated hemoglobin; TG: triglycerides; TC: total cholesterol; HDL-c: high-density lipoprotein; LDL-c: low-density lipoprotein; CRP: C-Reactive Protein; VAT1: the narrower region between the iliac crest and the last rib; VAT2: the midpoint between the last rib and the iliac crest; VAT3: 1 cm above the umbilical scar; SAT1: the narrower region between the iliac crest and the last rib; SAT2: the midpoint between the last rib and the iliac crest; SAT3: 1 cm above the umbilical scar.

There was no considerable superiority in the correlations between the means of AC and biochemical parameters when compared with the equivalent correlations of image measurements of VAT and SAT for both sexes. Likewise, relatively similar performances were observed between all correlations of AC measurements and biochemical variables ( Table 6 ).

**Table 6:** Pearson’s correlation (r) or Spearman’s ( *rho* ) correlation between averages of abdominal circumference (AC) measured in seven anatomical sites with biochemical parameters in adult individuals, according to sex

Variables	Males (n = 17)						
	AC1	AC2	AC3	AC4	AC5	AC6	AC7
FPG b	0.054	0.034	0.051	0.048	0.064	0.039	0.027
HbA1C b	0.641 *	0.641 *	0.655 *	0.655 *	0.655 *	0.606 *	0.606 *
TG b	0.488 *	0.498 *	0.510 *	0.504 *	0.493 *	0.495 *	0.488 *
TC a	0.249	0.269	0.255	0.265	0.283	0.280	0.276
HDL-c a	-0.575 *	-0.566 *	-0.556 *	-0.578 *	-0.577 *	-0.572 *	-0.563 *
LDL-c a	0.377	0.397	0.376	0.391	0.403	0.404	0.396
CRP b	0.735 *	0.735 *	0.756 *	0.758 *	0.785 **	0.788 **	0.800 **

Variables	Females (n=62)						
	AC1	AC2	AC3	AC4	AC5	AC6	AC7
FPG b	0.236	0.231	0.191	0.181	0.190	0.193	0.200
HbA1C b	0.320 *	0.327 *	0.301 *	0.261	0.280 *	0.281 *	0.291 *
TG b	0.341 *	0.323 *	0.308 *	0.258 *	0.278 *	0.271 *	0.297 *
TC a	0.145	0.146	0.139	0.121	0.120	0.115	0.104
HDL-c a	-0.338 *	-0.324 *	-0.318 *	-0.310 *	-0.295 *	-0.299 *	-0.292 *
LDL-c a	0.166	0.163	0.163	0.168	0.150	0.146	0.132
CRP b	0.380 *	0.350 *	0.323 *	0.326 *	0.338 *	0.313 *	0.331 *

* p < 0.05.

** p < 0.001.

a Pearson’s correlation.

b Spearman’s correlation.

FPG: Fasting Plasma Glucose; HbA1C: glycated hemoglobin; TG: triglycerides; TC: total cholesterol; HDL-c: high-density lipoprotein; LDL-c: low-density lipoprotein; CRP: C-Reactive Protein; AC1: the narrower region between the iliac crest and the last rib; AC2: immediately below the bone landmark of the last rib; AC3: midpoint between the last rib and the iliac crest; AC4: 1 cm above the umbilical scar; AC5: at the umbilical scar level; AC6: immediately above the bone landmark of the iliac crest; AC7: in the region of largest abdominal circumference.

For men, both HbA1C, TG, and HDL-c correlated moderately with all AC measurements, with a positive correlation being expressed by the variables HbA1c and TG (r > 0.60; p < 0.05 and r > 0.40; p < 0.05) and an inverse correlation being expressed by the variable HDL-c (r > -0.50; p < 0.05). CRP showed a strong correlation with all AC measurements (r > 0.70; p < 0.05), with a slightly higher correlation when AC was measured in the region of largest abdominal circumference (r = 0.800; p < 0.001). For women, AC measurements in the upper abdomen expressed slightly higher correlations with biochemical parameters when compared to the AC measurements in other regions of the abdomen ( Table 6 ).

## DISCUSSION

The high intra- and inter-evaluator agreement identified during the evaluation of the reproducibility of VAT and SAT measurements obtained by USG confirms the good reproducibility of the method used as a reference standard in this study, reinforcing the potential of USG as a tracking tool for abdominal adiposity, expressing high accuracy and reproducibility. Despite this, it should be considered that CT and MRI are the gold standard methods for assessing intraabdominal fat ( 32 , 33 ). However, the applicability of these methods in clinical practice is limited, due to the high cost and unavailability of equipment. In this context, the USG has additional advantages, such as accessibility, low invasiveness, ease of execution ( 4 ), the possibility of scanning the abdominal region ( 5 ), and the ability to identify minimal changes in the abdominal adipose tissue compartments ( 34 ).

The difference found in the distribution pattern of the different abdominal adipose tissue compartments between men and women can be attributed to the biological constitution of each sex, which modulates how VAT and SAT are presented along the abdominal region ( 35 ). The tendency of males to have a higher level of VAT and the tendency of females to have a higher level of SAT has been previously reported by other researchers ( 9 , 35 ).

When evaluating the correlation between VAT measurements by USG at different regions and VAT values obtained by Computed Tomography (CT) in 304 Caucasian adults and elderly individuals of both sexes, researchers also highlighted that the VAT area was slightly larger in men than in women, while women presented a higher concentration of SAT ( 36 ).

The particularities in the distribution pattern of adipose tissue according to sex help to explain the increased cardiometabolic risk associated with males ( 35 ) and reinforce the importance of investigating the distribution of different adipose compartments rather than evaluating only general adiposity.

In the literature, many studies seek to standardize the positional parameters for obtaining AC by evaluating the correlation of different anatomical sites with VAT values. However, few so far have performed a scan of the abdominal region with an imaging method to better evaluate and understand the how the intra-abdominal adipose tissues are organized along the abdomen.

The results showed that for men, correlations between VAT and AC measurements remained strong regardless of the measurement site, which can be explained by the uniformity of adipose disposition along the abdomen in this sex. For women, the correlations varied according to the measurement site, with better performances of AC measurements in the upper abdomen, where the smallest abdominal perimeters were observed, reinforcing that this region is more sensitive to evaluate visceral fat and more appropriate for tracking cardiometabolic risk in females.

Seimon and cols. ( 10 ) similarly found strong correlations between VAT measured by magnetic resonance (MR) and AC measured at the region corresponding to the smaller waist and at the midpoint between the last rib and the iliac crest in obese women, besides finding weak correlation between VAT and AC measured at umbilical level. The researchers also highlighted additional advantages of measuring the region corresponding to the smaller waist, such as greater ease and speed in execution.

The correlations found between VAT and the biochemical variables included, except for plasma glucose, can be explained by the fact that the accumulation of oxidation products of free fatty acids and the presence of inflammatory mediators in tissues adjacent to the portal circulation dysregulate the lipid profile, increasing in serum triglycerides and LDL, as well as reducing HDL levels, besides favoring the occurrence of insulin resistance and the production of inflammatory biomarkers, such as CRP, which lead to the dysregulation of metabolic homeostasis, favoring cardiometabolic complications ( 2 , 37 ).

As in the present study, Bellan and cols. ( 38 ) evaluated the association between the VAT measured by USG and cardiometabolic risk variables, finding a strong and direct correlation of VAT with fasting and 2-h plasma glucose (r = 0.26, p < 0.001; r = 0.28, p < 0.0001, respectively), fasting and 2-h plasma insulin (r = 0.41, p < 0.0001 for both), homeostatic model assessment for insulin resistance (HOMA-IR; r = 0.42, p < 0.0001), Framingham cardiovascular score (r = 0.44, p < 0.0001), and vascular age (r = 0.30, p < 0.001).

A cohort of Chinese adults who also sought to evaluate the association of VAT with metabolic risk factors found a strong correlation of VAT with higher blood pressure (βmen = 3.99, P = 0.0002; βwomen = 6.46, P = 0.0002), higher triglycerides (βmen = 0.45, P < 0.0001; βwomen = 0.6, P < 0.0001), higher total cholesterol (βmen = 0.15, P = 0.02; βwomen = 0.37, P = 0.0002), and higher 2-h glucose levels (βmen = 0.68, P = 0.003; βwomen = 0.94, P < 0.0001) ( 39 ).

With regard to cardiometabolic complications, several investigations have indicated an association of VAT with increased risk of diabetes and prediabetes ( 39 , 40 ), dyslipidemias ( 39 ), hypertension ( 39 ), cardiovascular diseases ( 41 ), metabolic syndrome ( 42 ), nonalcoholic fatty liver disease ( 43 ), polycystic ovary syndrome ( 44 ), and other diseases.

Among men, the VAT measured in the upper abdominal regions showed significant correlations with all biochemical parameters except for fasting glucose, which reinforces the superiority of this region in predicting cardiometabolic alterations. The AC measurements in the upper abdomen expressed correlations with the biochemical parameters, presenting a slightly better performance when compared to the AC measurements in other regions of the abdomen for women.

Similar results were reported by Pinho and cols. ( 9 ), who reported that the AC measurements in the upper abdomen, especially in the smaller waist, were correlated with a greater amount of biochemical parameters when compared to the AC measurements in other regions of the abdomen.

The results found reinforce the role played by doubly indirect inference methods, specifically the AC measurement, in the prediction of VAT and cardiometabolic alterations, besides highlighting the relevance of AC measured in the smaller waist circumference. Thus, its use is suggested, as it is capable of providing useful estimates of the visceral adipose tissue content and of cardiometabolic risk. In addition, the use of an alternative, simple, accessible, and effective instrument for predicting VAT values enables the identification and early action in conditions related to the abnormal distribution of body fat that leads to health complications ( 12 ).

Some limitations need to be considered when interpreting the results presented. Participants were selected by voluntary adherence and a random sample was not used. The small number of participants and the heterogeneous distribution of the sample between men and women may limit the statistical power of the study and compromise its external validity. The isolated use of C-reactive protein to evaluate the inflammatory profile is also a limitation due to the nonspecificity of this parameter. In addition, it should be considered that a gold standard method was not adopted for the evaluations carried out in this investigation.

The prior evaluation of the calibration of the ultrasound procedure by intra- and inter-evaluators for the analysis of different adipose compartments, as well as the use of a non-invasive imaging method capable of performing a scan of the abdominal region and evaluating the performance of different anatomical sites in the abdominal region, are important aspects to be highlighted in this study.

In conclusion, this study showed that the pattern of abdominal fat distribution differed between sexes. Women concentrate more VAT in the abdominal region, delimited in the smaller waist circumference, while men concentrate VAT uniformly along the abdomen. The correlations between VAT measured by USG and cardiometabolic parameters were relatively stronger in the upper regions of the abdomen, which reinforces the superiority of this region in predicting cardiometabolic alterations in both sexes.

## References

[1] Ross R, Neeland JI, Yamashita S, Shai S, Seidell J, Magni P (2020). Waist circumference as a vital sign in clinical practice: A Consensus Statement from the IAS and ICCR Working Group on Visceral Obesity. Nat Rev Endocrinol.

[2] Piché M, Tchernof A, Després J (2020). Obesity Phenotypes, Diabetes, and Cardiovascular Diseases. Circ Res.

[3] Ponti F, Cinque AD, Fazio N, Napoli A, Guglielmi G, Bazzocchi A (2020). Ultrasound imaging, a stethoscope for body composition assessment. Quant Imaging Med Surg.

[4] Mauad FM, Chagas-Neto FA, Benedeti ACGS, Nogueira-Barbosa MH, Muglia VF, Carneiro AAO (2017). Reproducibility of abdominal fat assessment by ultrasound and computed tomography. Radiol Bras.

[5] Gishti O, Gaillard R, Durmus B, Abrahamse M, Beek EMVD, Hofman A (2015). BMI, total and abdominal fat distribution, and cardiovascular risk factors in school-age children. Pediatr Res.

[6] Störchle P, Müller W, Sengeis M, Ahammer H, Fürhapter-Rieger A, Bachl N (2017). Standardized Ultrasound Measurement of Subcutaneous Fat Patterning: high reliability and accuracy in groups ranging from lean to obese. Ultrasound Med Biol.

[7] Smith MK, Christianto E, Staynor JMD (2020). Obesity and visceral fat in Indonesia: an unseen epidemic? a study using iDXA and surrogate anthropometric measures. Obes Res Clin Pract.

[8] Hernández-Reyes A, Vidal Á, Moreno-Ortega A, Cámara-Martos F, Moreno-Rojas R (2020). Waist Circumference as a Preventive Tool of Atherogenic Dyslipidemia and Obesity-Associated Cardiovascular Risk in Young Adults Males: A Cross-Sectional Pilot Study. Diagnostics.

[9] Pinho CPS, Diniz AS, Arruda IKG, Leite APDL, Petribu MMV, Rodrigues IG (2018). Waist circumference measurement sites and their association with visceral and subcutaneous fat and cardiometabolic abnormalities. Arch Endocrinol Metab.

[10] Seimon RV, Wild-Taylor AL, Gibson AA, Harper C, McClintock S, Fernando HA (2018). Less Waste on Waist Measurements: Determination of Optimal Waist Circumference Measurement Site to Predict Visceral Adipose Tissue in Postmenopausal Women with Obesity. Nutrients.

[11] Chaves TO, Reis MS (2018). Abdominal Circumference or Waist Circumference?. Int J Cardiovasc Sci.

[12] Tutunchi H, Ebrahimi-Mameghani M, Ostadrahimi A, Asghari-Jafarabadi M (2020). What are the optimal cut-off points of anthropometric indices for prediction of overweight and obesity? Predictive validity of waist circumference, waist-to-hip and waist-to-height ratios. Health Promot Perspect.

[13] Petribu MMV, Guimarães FJSP, Cabral PC, Santos EMC, Diniz AS, Arruda IKG (2012). Desenvolvimento e validação de equação preditiva da gordura visceral em mulheres. Rev Bras Cineantropometria Desempenho Hum.

[14] Correio ESL, Gonçalves JP (2019). Atribuições da Vida Pessoal de Jovens Adultos Universitários e Interferência no Desempenho Acadêmico. Rev Ensino Educ Ciênc Hum.

[15] Maio MC, Monteiro S, Chor D, Faerstein E, Lopes CS (2005). Cor/raça no Estudo Pró-Saúde: resultados comparativos de dois métodos de autoclassificação no Rio de Janeiro, Brasil. Cad Saúde Pública.

[16] (2013). Sociedade Brasileira de Cardiologia. I Diretriz Brasileira de Prevenção Cardiovascular. Arq Bras Cardiol.

[17] Pinho CPS, Diniz AS, Arruda LKG, Coelho PC, Sequeira LAS (2013). Prevalência e fatores associados à obesidade abdominal em indivíduos na faixa etária de 25 a 59 anos do Estado de Pernambuco, Brasil. Cad Saúde Pública.

[18] (2001). International Physical Activity Questionnaire (IPAQ).

[19] Alves VV, Ribeiro LFP, Barros R, Gadelha SR, Santos SC (2011). Circumference measured at different sites of the trunk and cardiometabolic risk factors. Rev Bras Cineantropometria Desempenho Hum.

[20] World Health Organization (1998). Obesity: preventing and managing the global epidemic. Report of a WHO consultation on obesit.

[21] Lohman TG, Roche AF, Martorell R (1988). Anthropometric standardization reference manual.

[22] Wang J, Thornton JC, Bari S, Williamson B, Gallagher B, Heymsfield SB (2003). Comparisons of waist circumferences measured at 4 sites. Am J Clin Nutr.

[23] World Health Organization (2000). Technical Report Series no. 894.

[24] Yang C, Wang L (2017). Comparisons of Waist Circumference Measurements at Five Different Anatomical Sites in Chinese Children. Biomed Res Int.

[25] Wang C, Chang W, Huang C, Tsai M, Lu T, Chou E (2020). Associations between Central Obesity and Outcomes of Adult In-hospital Cardiac Arrest: A Retrospective Cohort Study. Sci Rep.

[26] National Institutes of Health (2000). The practical guide identification, evaluation, and treatment of overweight and obesity in adults.

[27] Koch E, Otárola A, Romero T, Manríquez L, Paredes M, Kirschbaum A (2006). Mediciones antropométricas y riesgo de sufrir un evento cardiovascular no fatal en población chilena. Resultados del proyecto San Francisco. Rev Chil Cardiol.

[28] Oh J, Kim S, Shin D, Park K, Park SW, Cho Y (2011). A Simple Ultrasound Correlate of Visceral Fat. Ultrasound Med Biol.

[29] Philipsen A, Jørgensen ME, Vistisen D, Sandbaek A, Almdal TP, Christiansen JS (2015). Associations between Ultrasound Measures of Abdominal Fat Distribution and Indices of Glucose Metabolism in a Population at High Risk of Type 2 Diabetes: The ADDITION-PRO Study. PLoS One.

[30] Bertoli S, Leone A, Vignati L, Spadafranca A, Bedogni G, Vanzulli A (2015). Metabolic correlates of subcutaneous and visceral abdominal fat measured by ultrasonography: a comparison with waist circumference. Nutr J.

[31] Leite CC, Matsuda D, Berg BLW, Cerri GG, Halpern A (2000). Correlação da medida de espessura intra-abdominal medida pela ultrassonografia com os fatores de risco cardiovascular. Arq Bras Endocrinol Metab.

[32] Ryo M, Kishida K, Nakamura T, Yoshizumi T, Funahashi T, Shimomura I (2014). Clinical significance of visceral adiposity assessed by computed tomography: A Japanese perspective. World J Radiol.

[33] Eloi JC, Epifanio M, Gonçalves MM, Pellicioli A, Vieira PFG, Dias HB (2017). Quantification of Abdominal Fat in Obese and Healthy Adolescents Using 3 Tesla Magnetic Resonance Imaging and Free Software for Image Analysis. PLoS One.

[34] Stigall AN, Evans KD, Tatarski R, Pargeon R, Spees R (2018). Abdominal Adiposity Measured by Sonography as a Tool for Determining Disease Risk. J Diagn Med Sonogr.

[35] Lee M, Fried SK (2017). Sex-dependent Depot Differences in Adipose Tissue Development and Function; Role of Sex Steroids. J Obes Metab Syndr.

[36] Pimanov S, Bondarenko V, Makarenko E (2020). Visceral fat in different locations assessed by ultrasound: correlation with computed tomography and cut-off values in patients with metabolic syndrome. Clin Obes.

[37] Ma S, Xi B, Yang L, Sun J, Zhao M, Bovet P (2020). Trends in the prevalence of overweight, obesity, and abdominal obesity among Chinese adults between 1993 and 2015. Int J Obes.

[38] Bellan M, Menegatti M, Ferrari C, Carnevale Schianca GP, Pirisi M (2018). Ultrasound-assessed visceral fat and associations with glucose homeostasis and cardiovascular risk in clinical practice. Nutr Metab Cardiovasc Dis.

[39] Lizhi T, Fang Z, Nanwei T (2016). The association of visceral adipose tissue and subcutaneous adipose tissue with metabolic risk factors in a large population of Chinese adults. Clin Endocrinol.

[40] Pandora LW, Edward JB, Donna LL, Marguerite JM, Steven EK, Wilfred YF (2013). Change in Visceral Adiposity Independently Predicts a Greater Risk of Developing Type 2 Diabetes Over 10 Years in Japanese Americans. Diabetes Care.

[41] Ladeiras-Lopes R, Sampaio F, Bettencourt N, Fontes-Carvalho R, Ferreira N, Leite-Moreira A (2017). The Ratio Between Visceral and Subcutaneous Abdominal Fat Assessed by Computed Tomography Is an Independent Predictor of Mortality and Cardiac Events. Rev Española Cardiología.

[42] Lee YH, Park J, Min S, Kang O, Kwon H, Oh S (2020). Impact of Visceral Obesity on the Risk of Incident Metabolic Syndrome in Metabolically Healthy Normal Weight and Overweight Groups: A Longitudinal Cohort Study in Korea. Korean Journal of Family Medicine.

[43] Tantanavipas S, Vallibhakara O, Sobhonslidsuk A, Phongkitkarun S, Vallibhakara SA, Promson K (2019). Abdominal Obesity as a Predictive Factor of Nonalcoholic Fatty Liver Disease Assessed by Ultrasonography and Transient Elastography in Polycystic Ovary Syndrome and Healthy Women. BioMed Res Intl.

[44] Delitala AP, Capobianco G, Delitala G, Cherchi PL, Dessole S (2017). Polycystic ovary syndrome, adipose tissue and metabolic syndrome. Arch Gynecol Obstet.

